# Association of metabolic syndrome with brain imaging and cognitive function in UK Biobank

**DOI:** 10.1002/alz.085544

**Published:** 2025-01-09

**Authors:** Danial Qureshi, Anya G Topiwala, Md Shehab Uddin Al‐Abid, Naomi Allen, Elzbieta Kuzma, Thomas J Littlejohns

**Affiliations:** ^1^ University of Oxford, Oxford, England United Kingdom; ^2^ University of Oxford, Oxford United Kingdom; ^3^ University of Hamburg, Hamburg Germany

## Abstract

**Background:**

Metabolic syndrome (MetS) has been previously associated with an increased risk of developing dementia. Exploring links between MetS, neuroimaging and cognitive function measures can offer insights into whether MetS adversely affects brain health prior to dementia onset. We sought to examine the association of MetS with brain structure and cognition.

**Methods:**

Participants were selected from the UK Biobank multi‐modal imaging study (2014‐ongoing). MetS was defined as the presence of ≥3 of the following components: elevated waist circumference, triglycerides, blood pressure, HbA1c, or reduced HDL‐cholesterol. Neuroimaging outcomes included total brain, grey/white matter, white matter hyperintensity (WMH) and hippocampal volume from T1/T2‐weighted magnetic resonance imaging (MRI), and cognitive tests measuring executive function, reasoning, memory, and processing speed. Cross‐sectional associations were assessed using linear regression adjusted for age, sex, ethnicity, education, socioeconomic status, household income, smoking status, alcohol intake, physical activity, *APOE*‐ε4 carrier status, and assessment centre. Neuroimaging analyses were further adjusted for imaging‐related confounders (head size, head position, head motion).

**Results:**

Among 32,864 dementia‐free participants, 9,484 (29%) had MetS. MetS was significantly associated with lower total brain volume (β:‐0.06, 95%CI: ‐0.08, ‐0.04), grey matter volume (β: ‐0.09, 95%CI: ‐0.11, ‐0.07), hippocampal volume (left = β: ‐0.02, 95%CI: ‐0.05, ‐0.00; right = β: ‐0.03, 95%CI: ‐0.05, 0.00), and greater WMH volume (β: 0.10, 95%CI: 0.08, 0.12). MetS participants performed significantly worse on cognitive tests of working memory (β: ‐0.11, 95%CI: ‐0.14, ‐0.08), verbal declarative memory (β: ‐0.10, 95%CI: ‐0.12, ‐0.07), processing speed (β: ‐0.09, 95%CI: ‐0.12, ‐0.06), verbal and numerical reasoning (β: ‐0.06, 95%CI: ‐0.08, ‐0.03), executive function (β: 0.05, 95%CI: 0.02, 0.07), and non‐verbal reasoning (β: ‐0.04, 95%CI: ‐0.06, ‐0.01). We observed dose‐response relationships, with an increasing number of MetS components being associated with lower brain volume, greater WMH volume, and poorer cognitive function across all tests.

**Conclusion:**

MetS was associated with lower brain volume, greater vascular pathology, and poorer performance across multiple domains of cognitive function. These findings demonstrate that MetS is related to poorer brain health in dementia‐free individuals and could represent a novel risk factor for future development of dementia.

